# Improved Chondrogenic Differentiation of rAAV SOX9-Modified Human MSCs Seeded in Fibrin-Polyurethane Scaffolds in a Hydrodynamic Environment

**DOI:** 10.3390/ijms19092635

**Published:** 2018-09-05

**Authors:** Jagadeesh K. Venkatesan, Oliver Gardner, Ana Rey-Rico, David Eglin, Mauro Alini, Martin J. Stoddart, Magali Cucchiarini, Henning Madry

**Affiliations:** 1 Center of Experimental Orthopaedics, Saarland University, D-66421 Homburg/Saar, Germany; jegadish.venki@gmail.com (J.K.V.); Ana.Rey.Rico@gmail.com (A.R.-R.); mmcucchiarini@hotmail.com (M.C.); 2 AO Research Institute Davos, 7270 Davos Platz, Switzerland; olly1001@hotmail.com (O.G.); david.eglin@aofoundation.org (D.E.); mauro.alini@aofoundation.org (M.A.); martin.stoddart@aofoundation.org (M.J.S.); 3 Department of Orthopaedic Surgery, Saarland University Medical Center and Saarland University, D-66421 Homburg/Saar, Germany

**Keywords:** cartilage repair, hMSCs, chondrogenesis, rAAV, SOX9, fibrin-polyurethane scaffolds, bioreactors

## Abstract

The repair of focal articular cartilage defects remains a problem. Combining gene therapy with tissue engineering approaches using bone marrow-derived mesenchymal stem cells (MSCs) may allow the development of improved options for cartilage repair. Here, we examined whether a three-dimensional fibrin-polyurethane scaffold provides a favorable environment for the effective chondrogenic differentiation of human MSCs (hMSCs) overexpressing the cartilage-specific SOX9 transcription factor via recombinant adeno-associated virus (rAAV) -mediated gene transfer cultured in a hydrodynamic environment *in vitro*. Sustained SOX9 expression was noted in the constructs for at least 21 days, the longest time point evaluated. Such spatially defined SOX9 overexpression enhanced proliferative, metabolic, and chondrogenic activities compared with control (reporter *lacZ* gene transfer) treatment. Of further note, administration of the SOX9 vector was also capable of delaying premature hypertrophic and osteogenic differentiation in the constructs. This enhancement of chondrogenesis by spatially defined overexpression of human SOX9 demonstrate the potential benefits of using rAAV-modified hMSCs seeded in fibrin-polyurethane scaffolds as a promising approach for implantation in focal cartilage lesions to improve cartilage repair.

## 1. Introduction

Articular cartilage is the tissue that allows for a smooth, frictionless weightbearing surface in articulating joints. Once damaged, in the absence of vascularization and potentially regenerative cells, it has a limited ability for self-repair [1]. Although different surgical treatments are available, none of them permits a complete and long-lasting articular cartilage regeneration in adults [2,3] representing a particularly critical problem for orthopaedic surgeons.

Mesenchymal stem cells (MSCs) from the subchondral bone marrow are an attractive source of regenerative cells that might be employed to enhance articular cartilage repair [4]. Mesenchymal stem cells have a reliable potential for self-renewal and can differentiate into various cell lineages, among which the chondrocyte [5,6,7]. Mesenchymal stem cells have been already safely tested in clinical protocols as a means to treat articular cartilage defects and osteoarthritis [8,9]. The high density, aggregate culture system is a well-accepted model to evaluate MSC chondrogenesis [10]. However, this small experimental system is not best suited to assess cartilage tissue neoformation, repair, and extracellular matrix deposition. Also, even though some groups reported evidence showing the possibility of mechanically stimulating such aggregate cultures [11], their dimension is hindering the precise characterization of the effects of various mechanical loads. In this regard, fibrin-polyurethane composite scaffolds may provide a more adapted environment for MSC chondrogenesis as such biomaterials can provide mechanical stiffness that remains unaffected upon loading to preserve the MSC phenotype while serving as analogs of the natural extracellular matrix and providing extra, beneficial cues for cell differentiation [12,13,14]. While improved clinical parameters were frequently reported upon application of MSCs, without adverse reactions, such approaches thus far led to the production of a repair tissue of lesser quality relative to the original hyaline cartilage. To overcome such limitations, gene transfer combined with tissue engineering may allow to provide reparative signals in a spatially defined fashion [15,16] to increase the chondrogenic capacities of MSCs aiming at enhancing focal cartilage repair [17,18]. 

The sex-determining region Y-type high-mobility group box SOX9 transcription factor is a potent candidate to enhance MSC chondrogenesis. It plays a key role in cartilage formation [19,20] while delaying terminal differentiation and hypertrophy [20,21,22]. Gene transfer of SOX9 with recombinant adeno-associated viral (rAAV) vectors which are clinically adapted constructs provides for a safe profile in absence of viral coding sequences in their genome, allowing for high and particularly well-maintained levels of transgene expression in target cells among which MSCs [23,24,25,26,27]. Previously, rAAV was shown to successfully modify hMSCs via direct SOX9 gene transfer, leading to increased chondrogenic differentiation in vitro [27].

Here, we tested the hypothesis that administration of an rAAV SOX9 gene vector enhances the chondrogenic processes in hMSCs seeded in fibrin-PU scaffolds in bioreactors that provide a defined hydrodynamic environment in vitro. The results demonstrate that hMSCs can be modified via rAAV to overexpress SOX9 over an extended period of time within PU scaffolds, leading to an improved cell chondrogenic differentiation in such an environment relative to control (*lacZ*) vector treatment, as a promising future approach for the treatment of sites of cartilage injury.

## 2. Results

### 2.1. rAAV-Mediated SOX9 Overexpression in Human Mesenchymal Stem Cells Seeded in Polyurethane Scaffolds in a Hydrodynamic Environment

**Figure 1:**
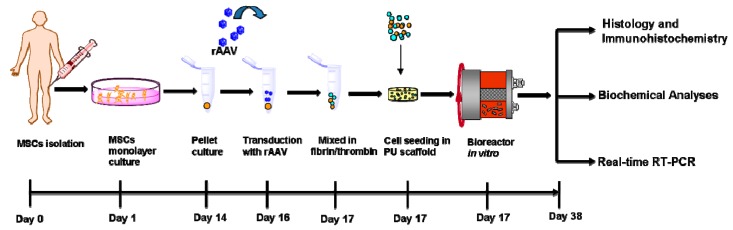
Model system: Human bone marrow-derived mesenchymal stem cells (hMSCs) were isolated from bone marrow aspirates, placed in monolayer culture, released, and hMSC aggregate cultures (2 × 10^5^ cells/pellet) were prepared and next transduced with rAAV as described in Materials and Methods. After 24 h, the transduced cells were placed in a fibrin/thrombin mixture and seeded onto fibrin-polyurethane (PU) scaffolds. The constructs were next transferred to rotating bioreactors and maintained in chondrogenic medium in hydrodynamic culture for 21 days for further evaluations as depicted.

Human adult mesenchymal stem cell (hMSC) aggregate cultures were first transduced with the candidate recombinant adeno-associated virus (rAAV) FLAG-tagged SOX9 (rAAV-FLAG-h*sox9*) vector compared with control (reporter rAAV-*lacZ* vector) treatment in order to evaluate whether rAAV was capable of promoting the overexpression of the transcription factor upon seeding of the modified cells in fibrin-polyurethane (PU) scaffolds and cultivation in hydrodynamic culture conditions in chondrogenic differentiation medium over time in vitro (Figure 1).

**Figure 2:**
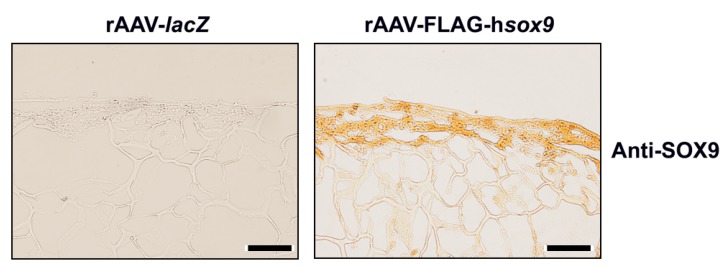
Detection of transgene (SOX9) expression in hydrodynamic cultures of rAAV-transduced hMSCs seeded in fibrin-PU scaffolds. Cells were transduced with rAAV-*lacZ* or rAAV-FLAG-h*sox9* (40 µL each vector), seeded in PU scaffolds, and cultivated in rotating bioreactors for 21 days using chondrogenic medium as described in Figure 1 and in Materials and Methods. Samples were processed after bioreactor cultivation to detect immunoreactivity to SOX9 (magnification ×10; scale bar: 200 µm; representative data). Note the highest presence of immunoreactivity to SOX9 at the surface of the constructs which can be attributed to a diffusion gradient of the nutrients into the center of the constructs at homogenous cell seeding.

Sustained SOX9 expression was seen in the SOX9-treated cells in PU scaffolds after 21 days of hydrodynamic culture stimulation relative to *lacZ* transduction (Figure 2). Specific SOX9 immunostaining was mostly observed at the surface of the constructs. A histomorphometric analysis performed using a system that grades the intensity of SOX9 immunostaining [28] revealed higher scores of SOX9 expression upon rAAV-FLAG-h*sox9* treatment compared with rAAV-*lacZ* (3.5-fold difference, *p* = 0.008) (Table 1).

### 2.2. Effects of SOX9 Overexpression upon the Biological and Chondrogenic Activities of hMSCs Seeded in PU Scaffolds in a Hydrodynamic Environment

The candidate SOX9 vector was then provided to hMSCs to monitor the effects of the transcription factor via rAAV application upon the biological and differentiation activities of the cells seeded in PU scaffolds and maintained in hydrodynamic culture conditions over time versus control *lacZ* gene transfer.

**Figure 3:**
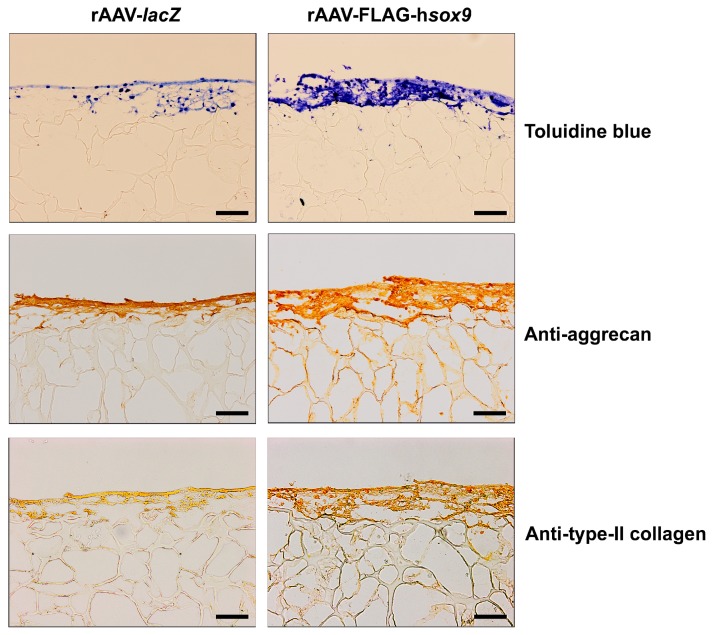
Histological analysis in hydrodynamic cultures of rAAV-transduced hMSCs seeded in fibrin-PU scaffolds. Cells were transduced, seeded in PU scaffolds, and placed in rotating bioreactors as described in Figure 1 and Figure 2 and in Materials and Methods. The samples were processed after 21 days for histological staining with toluidine blue and to detect immunoreactivity to aggrecan and to type-II collagen (magnification ×10; scale bar: 200 µm; all representative data). Similar to the immunoreactivity to SOX9, the high signals at the surface of the constructs can be attributed to a diffusion gradient of the nutrients into the center of the constructs at homogenous cell seeding.

**Figure 4:**
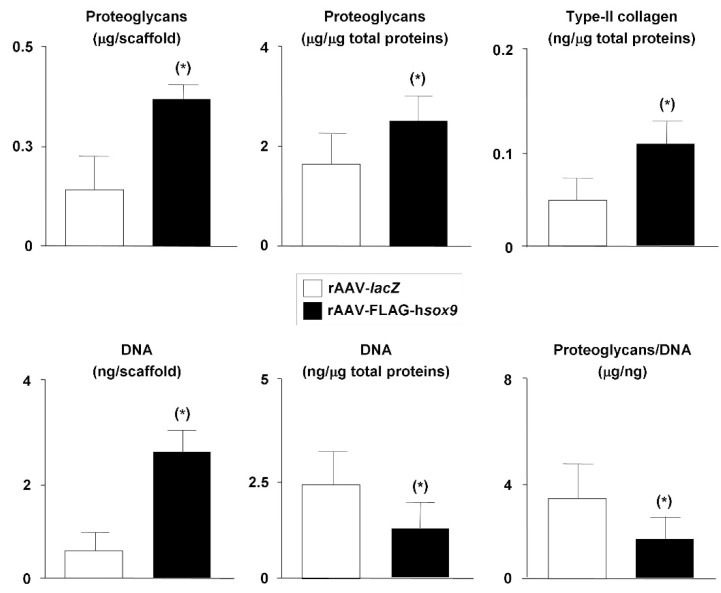
Biochemical analyses in hydrodynamic cultures of rAAV-transduced hMSCs seeded in fibrin-PU scaffolds. Cells were transduced, seeded in PU scaffolds, and placed in rotating bioreactors as described in the Figure 1, Figure 2 and Figure 3 and in Materials and Methods. The samples were processed after 21 days to monitor the proteoglycan contents by dimethylmethylene blue dye method, the type-II collagen contents by ELISA, and the DNA contents using Hoechst 33258 in the constructs. * Statistically significant compared with rAAV-*lacZ*.

Successful chondrogenic differentiation was noted in all treated samples after 21 days as seen by intense, more homogeneous toluidine blue staining, aggrecan and type-II collagen immunostaining but with higher staining intensities in the presence of the SOX9 vector (Figure 3). Specific toluidine blue staining, aggrecan, and type-II collagen immunostaining was mostly detected at the surface of the constructs. A histomorphometric analysis performed using a system that grades the intensity of toluidine blue staining and aggrecan or type-II collagen immunostaining [28] revealed higher scores of proteoglycan, aggrecan, and type-II collagen expression upon rAAV-FLAG-h*sox9* treatment compared with rAAV-*lacZ* (1.4-, 2.6-, and 2.7-fold difference, respectively; *p* ≤ 0.04) (Table 1). In good agreement, administration of rAAV-FLAG-h*sox9* significantly increased the proteoglycan and type-II collagen contents in the samples relative to rAAV-*lacZ* (1.7- and 10-fold, respectively; *p* ≤ 0.001) (Figure 4). Also of note, treatment with SOX9 significantly increased the DNA contents compared with *lacZ* (6.3-fold; *p* ≤ 0.001) (Figure 4).

### 2.3. Effects of SOX9 Overexpression upon the Hypertrophic and Osteogenic Activities of hMSCs Seeded in PU Scaffolds in a Hydrodynamic Environment

Finally, the candidate SOX9 vector was added to hMSCs to detect a potential influence of the transcription factor via rAAV application on the hypertrophic and osteogenic differentiation activities of the cells seeded in PU scaffolds and maintained in hydrodynamic culture conditions over time *versus* control *lacZ* gene transfer.

**Figure 5:**
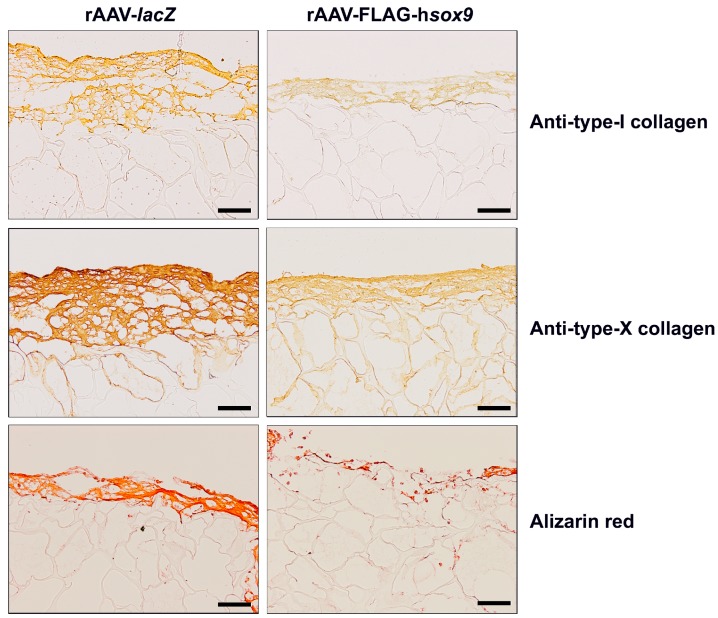
Immunohistochemical analysis in hydrodynamic cultures of rAAV-transduced hMSCs seeded in fibrin-PU scaffolds. Cells were transduced, seeded in PU scaffolds, and placed in rotating bioreactors as described in the Figure 1, Figure 2, Figure 3 and Figure 4 and in Materials and Methods. The samples were processed after 21 days to detect immunoreactivity to type-I and -X collagen and for histological staining with alizarin red (magnification ×10; scale bar: 200 µm; all representative data). Note the high signals at the surface of the constructs as a result of a diffusion gradient of the nutrients.

Remarkably, application of the rAAV SOX9 vector reduced the intensities of type-I and -X collagen immunostaining relative to control *lacZ* treatment (Figure 5). Specific type-I and -X collagen immunostaining was mostly seen at the surface of the constructs. A histomorphometric analysis performed using a system that grades the intensity of type-I and -X collagen immunostaining [28] revealed lower scores of type-I and -X collagen expression upon rAAV-FLAG-h*sox9* treatment compared with rAAV-*lacZ* (4- and 3.1-fold difference, respectively; *p* ≤ 0.046) (Table 1). Of further note, alizarin red staining intensities were less intense in the presence of SOX9 versus *lacZ* (Figure 5), with specific staining mostly detected at the surface of the constructs. These results are in good agreement with those reported by others and can indeed be attributed to the inadequate diffusion of the nutrients into the center of the constructs although cell seeding was homogenous [29]. The extracellular matrix developed along the cell-rich periphery is thought to interfere with the diffusion of the nutrients into the center of the scaffolds. A histomorphometric analysis performed using a system that grades the intensity of alizarin red staining [28] revealed lower scores of matrix mineralization upon rAAV-FLAG-h*sox9* treatment compared with rAAV-*lacZ* (3-fold difference; *p* = 0.008) (Table 1).

### 2.4. Real-Time RT-PCR Analyses in rAAV-Mediated SOX9-Overexpressing hMSCs Seeded in PU Scaffolds in a Hydrodynamic Environment

**Figure 6:**
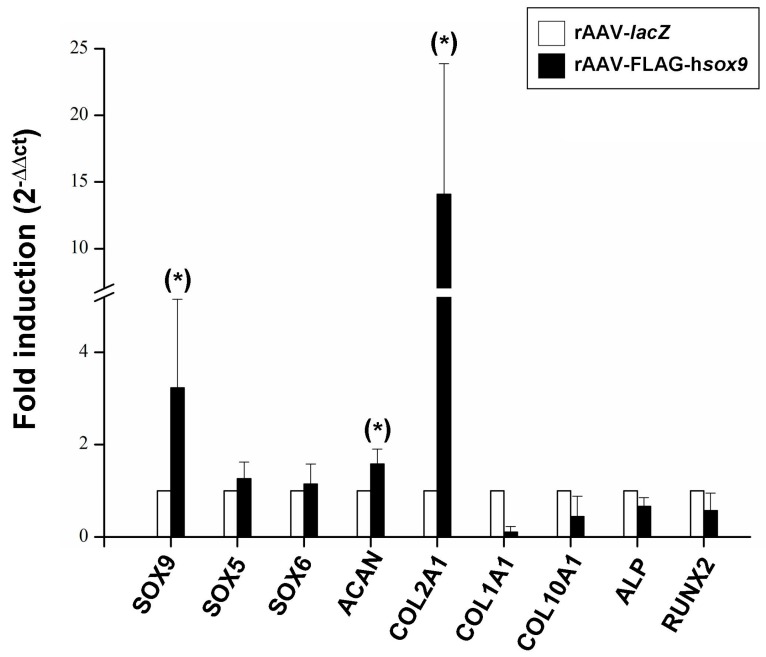
Real-time RT-PCR analysis in hydrodynamic cultures of rAAV-transduced hMSCs seeded in fibrin-PU scaffolds. Cells were transduced, seeded in PU scaffolds, and placed in rotating bioreactors as described in the Figure 1, Figure 2, Figure 3, Figure 4 and Figure 5 and in Materials and Methods. After 21 days of bioreactor cultivation, mRNA was directly isolated from the constructs of rAAV-transduced hMSCs in fibrin-PU scaffolds and the gene expression profiles of SOX9, SOX5, SOX6, aggrecan (ACAN), type-II (COL2A1), type-I (COL1A1), type-X collagen (COL10A1), alkaline phosphatase (ALP), and runt-related transcription factor 2 (RUNX2) were monitored, with GAPDH serving as a housekeeping gene and internal control (all primers are listed in Materials and Methods). Ct values were obtained for each target and for GAPDH as a control for normalization, and fold inductions (relative to *lacZ*-treated samples) were measured by using the 2^−ΔΔ*C*t^ method. * Statistically significant compared with rAAV-*lacZ*.

Overall, these findings were corroborated by results of a real-time RT-PCR analysis evaluating the gene expression profiles in the constructs. There was an enhanced chondrogenic differentiation and reduced osteogenic/hypertrophic differentiation of the cells in the presence of rAAV-FLAG-h*sox9* compared with rAAV-*lacZ* with significant increases observed in (~2.4-SOX9, 1.6-aggrecan, and 1.4-fold increased, type-II collagen expression levels *p* ≤ 0.001). Additionally, there was a trend towards an increased expression of SOX5 (1.26-fold) and SOX6 (1.14-fold) and ~10- and 2.4-fold decreased type-I and type-X collagen expression levels (Figure 6). A trend toward decreased expression profiles was also noted for alkaline phosphatase (ALP) and runt-related transcription factor 2 (RUNX2) in the presence of the rAAV SOX9 vector versus *lacZ* condition (1.5- and 1.7-fold decrease respectively; *p* = 0.094) (Figure 6).

## 3. Discussion

Induction of articular cartilage regeneration is one of the most challenging clinical problems on orthopaedic surgery. Combining gene therapy and tissue engineering approaches may provide effective, new workable procedures to improve the natural repair processes in sites of lesions [15] . While MSCs have been already employed in clinical implantation settings for cartilage repair [8,9], the outcomes have not met thus far the standards of regeneration. Directing MSCs toward an enhanced chondrogenic profile based on therapeutic gene transfer might be a valuable strategy to improve the processes of tissue healing [17] especially when providing the modified reparative cells within a biocompatible scaffold that supports cell repopulation [18].

In the present study, we evaluated the possibility of generating hydrodynamically [15] and chondrogenically adapted constructs by seeding rAAV SOX9-treated hMSCs in fibrin-PU scaffolds in light of the chondrogenic properties of the candidate transcription factor [19,20]. We employed the safe, clinically adapted rAAV vectors that do not impair the cell potency [23,24,25,26,27,30] and scaffolds known to provide a proper environment for MSC chondrogenesis [12,13,14]. Gene transfer of SOX9 to date has been performed using both nonviral [31,32], adenoviral [12,33], and retro-/lentiviral vectors [34,35]. Yet, such gene vehicles usually display low or short-term gene transfer efficiencies in cells undergoing proliferation (nonviral, adenoviral vectors) or may carry a risk for insertional mutagenesis (retro-/lentiviral vectors). Recombinant adeno-associated viral (rAAV) vectors instead are clinically adapted constructs, with a safe profile in absence of viral coding sequences in their genome, allowing for high and particularly well-maintained levels of transgene expression in their targets among which MSCs [23,24,25,26,30].

The data first indicate that hMSCs can be modified via rAAV gene transfer to overexpress SOX9 over 21 days within PU scaffolds in vitro extends previous work in three-dimensional hMSC aggregates in static culture [27]. Effective, sustained rAAV-mediated SOX9 gene transfer and expression was capable of durably stimulating the metabolic and chondrogenic activities (production of proteoglycans and type-II collagen) of hMSCs within PU scaffolds under hydrodynamic culture stimulation for at least 21 days compared with control treatment. This is in good agreement with the properties of the transcription factor [20,27,32,34,35] and with findings using SOX9-treated cells in the same scaffolds under stimulation in a custom-made bioreactor via adenoviral gene transfer [12] or in other types of biomaterials (alginate, polyglycolic acid) [31,33]. Such effects were accompanied by increases in SOX5 and SOX6 expression as noted when applying SOX9 to hMSCs via adenoviral gene transfer [12]. For comparison, Neumann et al. [14] reported that seeding of hMSCs modified by adenoviral vectors to produce BMP-2 in PU scaffolds resulted instead in a trend toward glycosaminoglycan/DNA ratios under hydrodynamic culture stimulation.

Remarkably, application of the rAAV SOX) vector also led to a trend towards a prolonged, advantageous reduction of undesirable osteogenic and hypertrophic expression profiles (type-I and -X collagen, matrix mineralization) in cells within the constructs. This is possibly due to decreased expression of the osteogenic markers ALP and RUNX2 (even though only a trend was observed under the conditions applied here). This is similar to the reported effects of SOX9 on bone formation, terminal differentiation, and calcification [21,22,36] and with previous findings, among which ours [20,27,32,33]. Interestingly, current treatment with rAAV SOX9 also led to enhanced levels of cell proliferation in contrast with our previous observations when the same vector was provided to hMSCs in aggregate cultures [27]. It is important to point out that in our previous report, rAAV SOX9 was directly applied to static cultures of scaffold-free hMSC cultures while cells here were seeded in a biocompatible scaffold submitted to hydrodynamic culture stimulation, a setting probably more favorable for cell division [37].

Of note, the effects reported here were mostly evidenced at the surface of the constructs where transgene expression was restricted, in contrast to previous evaluations using PU scaffolds [12,13]. Yet, in these earlier studies it is important to note that the cell-seeding densities were much higher than those applied here (2–5 × 10^6^ versus 2 × 10^5^ cells/scaffold here, i.e., 10- to 25-fold difference). An analysis is currently being performed to assess the impact of higher cell-seeding densities on the levels and significance of chondrogenic differentiation in the current system in order to further increase articular cartilage resurfacing and restore the integrity of damaged articular cartilage. Work is also ongoing to evaluate the potential of the approach in vivo by implanting similar constructs in experimental models of articular cartilage defects [15,20,33,35,38] in light of the performance of PU scaffolds in vivo [39]. Taken together, the present study demonstrates preliminary benefits of the propagation of constructs made of rAAV SOX9-transduced hMSCs in PU scaffolds in hydrodynamic culture conditions as a possible tool to generate adapted treatments for articular cartilage lesions.

## 4. Materials and Methods

### 4.1. Reagents

Reagents were from Sigma (Munich, Germany) unless otherwise indicated. Recombinant FGF-2 (rFGF-2) and TGF-β3 were purchased at R&D Systems (Wiesbaden-Nordenstadt, Germany) and Peprotech (Rocky Hill, NJ, USA), respectively. The dimethylmethylene blue dye was from Serva (Heidelberg, Germany). The anti-SOX9 (C-20) antibody was from Santa Cruz Biotechnology (Heidelberg, Germany), the anti-aggrecan (BC-13) antibody from Abcam (Cambridge, UK), the anti-type-II collagen (AF-5710) and anti-type-I collagen (AF-5610) antibodies from Acris (Hiddenhausen, Germany), the anti-type-X collagen (COL-10) antibody from Sigma, and biotinylated secondary antibodies with ABC reagent from Vector Laboratories (Alexis Deutschland GmbH, Grünberg, Germany). The type-II collagen enzyme-linked immunosorbent assay (Arthrogen-CIA Capture ELISA kit) was from Chondrex (Redmond, WA, USA).

### 4.2. Cell Culture

Bone marrow aspirates (~15 mL) were obtained from the distal femurs of patients undergoing total knee arthroplasty (*n* = 10). The study was approved by the Ethics Committee of the Saarland Physicians Council. All patients provided informed consent before inclusion in the study. All procedures were in accordance with the Helsinki Declaration. Human mesenchymal stem cells (hMSCs) were isolated and expanded in culture by using standard protocols [27]. Briefly, the aspirates were washed in DMEM and the cell-containing fractions layered onto Histopaque density gradient and centrifuged at 800× *g* for 30 min at room temperature. The nucleated cell fraction at the interface was collected, washed, and resuspended in Mesencult basal medium containing MSC stimulatory supplements (StemCell Technologies, Cologne, Germany) with 100 U/mL penicillin, 100 µL/mL streptomycin, and rFGF-2 (10 ng/mL). hMSCs were plated at 2 × 10^5^ cells/cm^2^ in T75 flasks and maintained at 37 °C in a humidified atmosphere with 5% CO_2_. The medium was exchanged after 48 h and every 2 to 3 days thereafter. The cells were detached and re-plated for further experiments at appropriate densities (2 × 10^5^ cells). hMSCs were analyzed with flow cytometry for expression of stem-cell surface markers (CD71^+^, CD105^+^, CD34^−^). All experiments were performed with cells at not more than passage two. Cells for all patients (*n* = 10) were tested in all the assays.

### 4.3. Plasmids and rAAV Vectors

The constructs were all derived from the same parental AAV-2 genomic clone—pSSV9 [40,41]. rAAV-*lacZ* is an AAV-2-based vector plasmid carrying the *lacZ* gene encoding β-galactosidase (β-gal) under the control of the cytomegalovirus immediate-early (CMV-IE) promoter [20,27]. rAAV-FLAG-h*sox9* is the same AAV-2-based vector plasmid used to prepare rAAV-*lacZ* but carrying a FLAG-tagged SOX9 sequence (1.7 kb) instead of *lacZ* [20,27]. All vectors were packaged as conventional (not self-complementary) vectors in the 293 cell line, an adenovirus-transformed human embryonic kidney cell line, by using Adenovirus 5 to provide helper functions in combination with the transacting AAV-2 factors for replication and encapsidation functions supplied by the pAd8 helper plasmid. The vector preparations were purified, dialyzed, and titered by real-time PCR [20,27], averaging 10^11^ functional units/mL.

### 4.4. rAAV-Mediated Gene Transfer and Hydrodynamic Bioreactor Culture

The hMSCs aggregate cultures (2 × 10^5^ cells) were prepared and kept in DMEM high glucose (4.5 g/L), 100 U/mL penicillin, 100 µL/mL streptomycin, ITS^+^ Premix (insulin 6.25 µg/mL, transferrin 6.25 µg/mL, selenous acid 6.25 µg/mL, linoleic acid 5.35 µg/mL, bovine serum albumin 1.25 µg/mL), pyruvate (1 mM), ascorbate 2-phosphate (37.5 µg/mL), dexamethasone (10^−^^7^ M), and TGF-β3 (10 ng/mL) (chondrogenic differentiation medium) at 37 °C in a humidified atmosphere with 5% CO_2_ [27]. The hMSC aggregate cultures were transduced with rAAV (40 µL vector, MOI = 4) or let untreated one day after aggregate formation [27]. A mixture of fibrinogen (17 mg/mL)/thrombin (5 U/mL) (Baxter, Volketswil, Switzerland) was then added to the cells that were then seeded in fibrin-polyurethane (PU) scaffolds (pore size: 90–300 µm) that allow for 100% yield of cell attachment, high initial cell density, and spatially uniform cell distribution [12,13,14]. The scaffolds were cultured in free suspension in flow rotating bioreactors under simulated microgravity and low shear stress (RCCV-110; Synthecon, Houston, TX) with an annular space between an outer cylinder and and inner gas exchange membrane that rotates around its axis to provide controlled hydrodynamic culture conditions optimal for chondrogenesis [15,42] for 21 days in chondrogenic differentiation medium for further evaluations (Figure 1).

### 4.5. Histology, Immunocytochemistry, and Immunohistochemistry

The constructs were harvested, fixed in 100% methanol, embedded in Jung tissue freezing compound, and cryosectioned at 12 µm [20,27]. Samples were processed for immunohistochemical analyses using specific antibodies, and sections were also stained with toluidine blue (matrix proteoglycans) and alizarin red (matrix mineralization) according to routine protocols [20,27]. Expression of aggrecan, type-II, -I, and -X collagen was detected by immunohistochemistry by using specific antibodies, biotinylated secondary antibodies, and the ABC method with diaminobenzidine (DAB) as the chromogen [20,27].

### 4.6. Histomorphometry

The immunohistochemical and histological grading scores were measured using four histological sections for each condition with the SIS AnalySIS program (Olympus, Hamburg, Germany). Toluidine blue and alizarin red staining and SOX9, type-II, -I, and -X collagen immunostaining were scored for uniformity and intensity according to a modified Bern Score grading system [28] as: 0 (no staining), 1 (heterogeneous and/or weak staining), 2 (homogeneous and/or moderate staining), 3 (homogeneous and/or intense staining), and 4 (very intense staining). Sections were scored blind by two individuals with regard to the conditions.

### 4.7. Biochemical Assays

Cultures were harvested with selective papain digestion from scaffolds. The DNA contents were determined with a fluorimetric assay by using Hoechst 33258, the proteoglycan contents by binding to dimethylmethylene blue dye, and the type-II collagen contents by ELISA [20,27]. All measurements were performed using a GENios spectrophotometer/fluorometer (Tecan, Crailsheim, Germany).

### 4.8. Real-Time RT-PCR Analyses

Total cellular RNA was extracted from the cultures by using the RNeasy Protect Mini Kit with an on-column RNase-free DNase treatment (Qiagen, Hilden, Germany). RNA was eluted in 30 µL RNase-free water. Reverse transcription was carried out with 8 µL of eluate by using the 1st Strand cDNA Synthesis kit for RT-PCR (AMV) (Roche Applied Science). An aliquot of the cDNA product (3 µL) was amplified with real-time PCR by using the Brilliant SYBR Green QPCR Master Mix (Stratagene, Agilent Technologies, Waldbronn, Germany) [27] on an Mx3000P QPCR operator system (Stratagene) as follows: (95 °C, 10 min), amplification by 55 cycles (denaturation at 95 °C, 30 s; annealing at 55 °C, 1 min; extension at 72 °C, 30 s), denaturation (95 °C, 1 min), and final incubation (55 °C, 30 s). The primers (Invitrogen GmbH) used were SOX9 (chondrogenic marker) (forward 5′-ACACACAGCTCACTCGACCTTG-3′; reverse 5′-GGGAATTCTGGTTGGTCCTCT-3′), SOX5 (chondrogenic marker) (forward 5′-ATCCCAACTACCATGGCAGCT-3′; reverse 5′-GATACCTGCATTGCAGCT-3′), SOX6 (chondrogenic marker) (forward 5′-GCAGTGATCAACATGTGGCCT-3′; reverse 5′-TTCATCATGCGCTGCCAGTAG-3′), aggrecan (ACAN) (chondrogenic marker) (forward 5′-GAGATGGAGGGTGAGGTC-3′; reverse 5′-ACGCTGCCTCGGGCTTC-3′), type-II collagen (COL2A1) (chondrogenic marker) (forward 5′-GGACTTTTCTCCCCTCTCT-3′; reverse 5′-GACCCGAAGGTCTTACAGGA-3′), type-I collagen (COL1A1) (osteogenic marker) (forward 5′-ACGTCCTGGTGAAGTTGGTC-3′; reverse 5′-ACCAGGGAAGCCTCTCTCTC-3′), type-X collagen (COL10A1) (marker of hypertrophy) (forward 5′-CCCTCTTGTTAGTGCCAACC-3′; reverse 5′-AGATTCCAGTCCTTGGGTCA-3′), alkaline phosphatase (ALP) (osteogenic marker) (forward 5′-TGGAGCTTCAGAAGCTCAACACCA-3′; reverse 5′-ATCTCGTTGTCTGAGTACCAGTCC-3′), runt-related transcription factor 2 (RUNX2) (osteogenic marker) (forward 5′-GCAGTTCCCAAGCATTTCAT-3′; reverse 5′-CACTCTGGCTTTGGGAAGAG-3′), and glyceraldehyde-3-phosphate dehydrogenase (GAPDH) (housekeeping gene and internal control) (forward, 5′-GAAGGTGAAGGTCGGAGTC-3′; reverse, 5′-GAAGATGGTGATGGGATTTC-3′) (all 150 nM final concentration) [27]. Control conditions included reactions using water and nonreverse-transcribed mRNA. Specificity of the products was confirmed by melting curve analysis and agarose gel electrophoresis. The threshold cycle (Ct) value for each gene of interest was measured for each amplified sample using MxPro QPCR software (Stratagene), and values were normalized to GAPDH expression by using the 2^−ΔΔ*C*t^ method, as described previously [27].

### 4.9. Statistical Analysis

Data are expressed as mean ± standard deviation (SD) of separate experiments. Each treatment condition was performed in quadruplicate in two independent experiments for each patient. All patients (*n* = 10) were tested in the assays. Data were obtained by two individuals that were blinded with respect to the treatment groups. The *t* test and the Mann–Whitney Rank Sum Test were used where appropriate. Any *p* value of less than 0.05 was considered statistically significant.

## 5. Conclusions

Chondrogenic differentiation of hMSCs genetically modified via rAAV to overexpress SOX) and seeded in PU scaffolds undergo enhanced chondrogenic differentiation in hydrodynamic culture conditions. This combined cell, gene, and scaffold approach may find value in developing novel treatments for articular cartilage defects.

## Figures and Tables

**Table 1:** Effects of SOX9 overexpression on the histomorphometry of the fibrin-PU scaffolds seeded with rAAV-transduced hMSCs.

Parameter	rAAV-*lacZ*	rAAV-FLAG-h*sox9*
SOX9 immunostaining	0.8 (0.5)	2.8 (0.5) *
Toluidine blue staining	1.8 (0.5)	2.5 (0.6) *
Aggrecan immunostaining	0.8 (0.3)	2.1 (0.6) *
Type-II collagen immunostaining	0.9 (0.1)	2.4 (0.2) *
Type-I collagen immunostaining	2.4 (0.5)	0.6 (0.2) *
Type-X collagen immunostaining	4.0 (0.1)	1.3 (0.5) *
Alizarin red staining	3.3 (0.5)	1.1 (0.5)*

Toluidine blue and alizarin red staining and SOX9, type-II, -I, and -X collagen immunostaining were scored for uniformity and intensity as: 0 (no staining), 1 (heterogeneous and/or weak staining), 2 (homogeneous and/or moderate staining), 3 (homogeneous and/or intense staining), and 4 (very intense staining) [28]. Values are given as mean (SD; *n* = 4). * Statistically significant compared with rAAV-*lacZ*.
